# Haematological and immunological data of Chinese children infected with coronavirus disease 2019

**DOI:** 10.1016/j.dib.2020.105953

**Published:** 2020-06-30

**Authors:** Xiaoli Xiong, Gilbert T. Chua, Shuiqing Chi, Mike Yat Wah Kwan, Wilfred Hing Sang Wong, Aifen Zhou, Chi Chiu Shek, Keith T.S. Tung, Huan Qin, Rosa S. Wong, Xue Li, Peng Chen, Shuai Li, Celine S. Chui, Winnie W.Y. Tso, Marco H.K. Ho, Ian C.K. Wong, Godfrey C.F. Chan, Yu Lung Lau, Kenneth K.Y. Wong, Patrick H.Y. Chung, Hui Li, Paul K.H. Tam, Shao-Tao Tang, Patrick lp

**Affiliations:** a Department of Integrated Chinese and Western Medicine, Wuhan Children's Hospital (Wuhan Maternal and Child Healthcare Hospital), Tongji Medical College, Huazhong University of Science and Technology, Wuhan, China; b Department of Paediatrics and Adolescent Medicine, The University of Hong Kong, Hong Kong SAR, China; c Department of Pediatric Surgery, Union Hospital, Tongji Medical College, Huazhong University of Science and Technology, Wuhan, China; d Department of Paediatrics and Adolescent Medicine, Princess Margaret Hospital, Hong Kong SAR, China; e Department of Maternal Healthcare, Wuhan Children's Hospital (Wuhan Maternal and Child Healthcare Hospital), Tongji Medical College, Huazhong University of Science and Technology, Wuhan, China; f Institute of Maternal and Child Health, Wuhan Children's Hospital (Wuhan Maternal and Child Healthcare Hospital), Tongji Medical College, Huazhong University of Science and Technology, Wuhan, China; g Centre for Safe Medication Practice and Research, Department of Pharmacology and Pharmacy, The University of Hong Kong, Hong Kong SAR, China; h Department of Respiratory Medicine, Wuhan Children's Hospital (Wuhan Maternal and Child Healthcare Hospital), Tongji Medical College, Huazhong University of Science and Technology, Wuhan, China; i Research Department of Practice and Policy, UCL School of Pharmacy, University College, London, United Kingdom; j Division of Paediatric Surgery, Department of Surgery, The University of Hong Kong, Hong Kong SAR, China; k Department of Hematology, Wuhan Children's Hospital (Wuhan Maternal and Child Healthcare Hospital), Tongji Medical College, Huazhong University of Science and Technology, Wuhan, China; l Dr. Li Dak Sum Research Centre, The University of Hong Kong-Karolinska Institutet Collaboration in Regenerative Medicine, The University of Hong Kong China

**Keywords:** COVID-19, Children, Chinese, Lymphocyte subsets, Cytokines, Immunoglobulin

## Abstract

Haematological and immunological data of children with COVID-19 infection is lacking. Between 21st January and 20th March 2020, 244 children who were confirmed to have COVID-19 infection and admitted to the Wuhan Children's Hospital, China were retrospectively reviewed. 193 children were considered as symptomatic, which was defined as having either the presence of clinical symptoms or the presence of CT thorax abnormalities. Their haematological and immunological profiles, including complete blood counts, lymphocyte subsets (T, B and NK cell counts), immunoglobulin (Ig) profiles (IgG, IgA and IgM) and cytokine profiles were analysed and compared between the symptomatic and asymptomatic groups. The median values and the interquartile ranges were calculated. Comparison was made using the Mann–Whitney U test. Children with symptomatic COVID-19 infection had significantly lower haemoglobin levels, but higher absolute lymphocyte and monocyte counts, IgG and IgA levels, as well as interleukin 6 (IL-6), IL-10, tumour necrosis factor alpha and interferon gamma levels. The obtained data will be utilized for further studies in comparing children and adults with COVID-19 infections in other parts of the world and with different severity .

Specifications table

**Table:** 

Subject	Infectious disease
Specific subject area	Immunological profiles of children with COVID-19 infection
Type of data	Table
How data were acquired	Children in Wuhan, China who were tested positive for SARS-CoV-2 by nasopharyngeal aspirate (NPA) reverse-transcriptase polymerase chain reactions (RT-PCR) were admitted to the Wuhan Children's Hospital between 21st January and 20th March 2020. Their hospital records and laboratory results were retrieved and analysed.
Data format	Raw
Parameters for data collection	Age, gender, complete blood counts, lymphocyte subset profiles, immunoglobulin profiles and cytokine profiles
Description of data collection	Laboratory data were retrospectively retrieved from the patients’ hospital record.
Data source location	Institution: Wuhan Children's Hospital City/Town/Region: Wuhan Country: China
Data accessibility	With the article
Related research article	Xiong X, Chua GT, Chi S, et al. A Comparison Between Chinese Children Infected with COVID-19 and with SARS. *J Pediatr* 2020 doi: https://doi.org/10.1016/j.jpeds.2020.06.041[1]

## Value of the data

• The COVID-19 pandemic has caused more than 5 million people being infected and 350,000 people died [2]. Although the majority of paediatric cases have been reported to have mild symptoms, their haematological and immunological profiles has not been well described.
• Understanding the differences in the haematological and immunological profiles between children with symptomatic and asymptomatic COVID-19 infections may help the utilization of targeted therapies in treatment COVID-19 infections, especially for severe cases.
• These data will also allow scientists and researchers to compare the immunological profiles of children with COVID-19 infection between different countries, especially with recent reports from Europe on the occurrence of Kawasaki-like syndrome, which was not observed in our Chinese cohort [3].

## 1 Data description

Children with COVID-19 infection confirmed by nasopharyngeal aspirate (NPA) SARS-CoV-2 reverse-transcriptase polymerase chain reaction (RT-PCR) were admitted to the Wuhan Children's Hospital between 21st January and 20th March 2020. Laboratory data were retrospectively retrieved from the patients’ hospital record. The age and gender, as well as their haematological and immunological profiles were recorded (supplementary file), including their complete blood counts (total white cell count, haemoglobin concentration, platelet counts, absolute neutrophil, lymphocyte and monocyte counts), lymphocyte subset profiles (total CD3 T cell count, CD3CD4 helper T cell count, CD3CD8 cytotoxic T cell count, CD19 B cell count and CD16CD56 natural killer cell count), immunoglobulin profiles (IgG, IgA and IgM) and cytokine profiles (interleukin 2 (IL-2), IL-4. IL-6, IL-10, tumour necrosis factor alpha and interferon gamma levels).

Table 1 compared the haematological and immunological profiles between children with symptomatic and asymptomatic COVID-19 infection. Children with symptomatic COVID-19 infection had significantly lower haemoglobin levels, but higher absolute lymphocyte and monocyte counts, IgG and IgA levels, as well as interleukin 6 (IL-6), IL-10, tumour necrosis factor alpha and interferon gamma levels.

**Table 1:** Immunological profiles of children with symptomatic and asymptomatic COVID-19 infection.

	Symptomatic (*n* = 193)			Asymptomatic (*n* = 51)			
	Median	Interquarile Range	Missing data	Median	Interquarile Range	Missing data	p-value
Total white cell count(× 10^9^/L)	6.76	5.37 - 8.27	10	6.41	5.36 - 7.16	4	0.15
Haemoglobin (g/L)	125	115 - 133	10	132	125 - 138	4	0.0003
Platelets (× 10^9^/L)	287	239 - 368	10	286	247 - 336	4	0.56
Absolute Neutrophil Count (× 10^9^/L)	2.36	1.72 - 3.49	10	3.12	2.26 - 3.69	4	0.05
Absolute Lymphocyte Count (× 10^9^/L)	2.97	1.97 - 4.56	9	2.67	1.86 - 3.08	4	0.04
Absolute Monocyte Count (× 10^9^/L)	0.43	0.35 - 0.59	10	0.39	0.32 - 0.48	4	0.02
Total T Cells (CD3) (cells/uL)	2246	1639 - 3197	38	1970	1677 - 2464	6	0.08
Helper T Cells (CD3CD4) (cells/uL)	1172	806 - 1820	76	1122	848 - 1395	23	0.4
Cytotoxic T Cells (CD3CD8) (cells/uL)	1006	680 - 1281	78	991	768 - 1183	23	0.87
B Cells (CD19) (cells/uL)	618	381 - 900	39	539	432 - 644	6	0.18
NK Cells (CD16/56) (cells/uL)	342	199 - 507	39	257	156 - 474	6	0.11
IgG (g/L)	9.19	6.14 - 11.2	14	10.05	8.75 - 11.65	3	0.004
IgA (g/L)	1.01	0.33 - 1.69	14	1.34	1.13 - 1.74	3	0.005
IgM (g/L)	0.9	0.6 - 1.18	14	0.89	0.69 - 1.33	3	0.39
IL2 (pg/ml)	1.43	1.23 - 1.71	36	1.33	1.15 - 1.56	4	0.1
IL4 (pg/ml)	2.66	2.12 - 3.25	36	2.46	1.95 - 3.24	4	0.19
IL6 (pg/ml)	3.98	2.95 - 7.25	36	3.53	2.59 - 4.36	4	0.01
IL10 (pg/ml)	3.85	3.23 - 5.16	36	3.08	2.70 - 3.45	4	<0.0001
Tumour necrosis factor-alpha (pg/ml)	1.7	1.24 - 2.20	36	1.41	1.15 - 1.89	4	0.04
Interferon-gamma (pg/ml)	3.01	2.39 - 4.25	36	2.46	1.85 - 3.38	4	0.004

## 2 Experimental design, materials and methods

Data were analysed using Microsoft Excel® (Redmond, Washington, USA) and GraphPad® Prism 8 (San Diego, California, USA). The median values and interquartile ranges were calculated. For comparison of data between the symptomatic and asymptomatic groups, the Mann–Whitney U test was used for continuous variables. A p-value of <0.05 is considered as statistically significant.

## Declaration of Competing Interest

The authors declare that they have no known competing financial interests or personal relationships which have, or could be perceived to have, influenced the work reported in this article.

## Appendix Supplementary materials

Image, application 1
